# Ultrasonography of herniated lumbar discs for screening programs in the late childhood and teenage

**DOI:** 10.1186/1878-5085-5-S1-A164

**Published:** 2014-02-11

**Authors:** Rizvan Ya Abdullaiev, Rostyslav V Bubnov, Ilgar G Mammadov, Ruslan R Abdullaiev

**Affiliations:** 1 Kharkiv Medical Academy of Postgraduate Education, Kharkiv, Ukraine; 2 Clinical Hospital ”Pheophania“ of State Affairs Department, Kyiv, Ukraine; 3 Kharkiv National MedicalUniversity, Kharkiv, Ukraine

## Scientific objectives

Lumbar spine disorders, mostly scoliosis are frequently diagnosed in childhood. Early detection of lumbar spine herniation makes possible to perform timely treatment and prophylaxys. Magnetic resonance imaging is basic method for spine diagnostics, however ultrasonography can become the relevant alternave for screening.

## Objective

To improve the efficiency of diagnosis of hernia of intervertebral discs (IVD) of the lumbar spine in children by determining the ultrasound biomarkers.

## Materials and methods

Ultrasound and X-ray study 178 children with low back pain observed in the pediatric neurologist and an orthopedic and trauma at the age of 13 to 18 years. All patients had clinical signs of degenerative disc disease. Ultrasonography of the lumbar spine held transabdominal approach on the levels of L1-L2 by L5-S1.

## The results

In 101 children changes in the IVD were registered within the nucleus pulposus: among them in 45 (44.6 ± 4.9%) aged 15-16 years, in 35 (34.6 ± 4.7%) in the age 13-14 years and 21 (20.8 ± 4.0%) in the age of 17-18 years. In these children we found an increased echogenicity and the displacement of nucleus pulposus. In 63 children than offset the nucleus pulposus had abnormal fibrous ring in the form of non-uniform thinning, increased echogenicity and posterior protrusion of up to 3 mm. Among them in 8 children (12.7 ± 4.2%) was within the age of 13-14 years, in 16 (25.4 ± 5.5%) - at 15-16 years and 39 (61.9 ± 6.1%) - at the age of 15-16 years. In 14 children we found mentioned discontinuous images of the fibrous ring of median or paramedian localizations in the nucleus pulposus protrusion more than 3 mm - in 11 (78.6 ± 11.0%) aged 17-18 years children and in 3 (21.4 ± 11.0%) - at the age of 15-16 years. Statistical analysis shown the disc herniation significantly more common among children aged 17-18 years (p <0.01 and p <0.05, respectively).

## Conclusion

The ultrasound is an effective method for diagnosis of disc herniation, which at the age of 17-18 years is significantly more common than in the younger groups.

## Outlook and expert recommendations

It is recommended to initiate ultrasound screening program for disc herniation in teenagers at National level; to organize educational programs for lumbar spine ultrasonography for radiologists to create screening network.

